# Innervation zones of fasciculating motor units: observations by a linear electrode array

**DOI:** 10.3389/fnhum.2015.00239

**Published:** 2015-05-12

**Authors:** Faezeh Jahanmiri-Nezhad, Paul E. Barkhaus, William Z. Rymer, Ping Zhou

**Affiliations:** ^1^Department of Bioengineering, University of Illinois at ChicagoChicago, IL, USA; ^2^Single Motor Unit Lab, Sensory Motor Performance Program, Rehabilitation Institute of ChicagoChicago, IL, USA; ^3^Department of Neurology, Medical College of Wisconsin and the Milwaukee Veterans Administration Medical CenterMilwaukee, WI, USA; ^4^Department of Physical Medicine and Rehabilitation, Northwestern UniversityChicago, IL, USA; ^5^Department of Physical Medicine and Rehabilitation, University of Texas Health Science Center and TIRR Memorial Hermann Research CenterHouston, TX, USA; ^6^Biomedical Engineering Program, University of Science and Technology of ChinaHefei, China

**Keywords:** innervation zone, ALS, fasciculation potentials, motor unit potentials, linear array EMG electrode, reinnervation

## Abstract

This study examines the innervation zone (IZ) in the biceps brachii muscle in healthy subjects and those with amyotrophic lateral sclerosis (ALS) using a 20-channel linear electromyogram (EMG) electrode array. Raster plots of individual waveform potentials were studied to estimate the motor unit IZ. While this work mainly focused on fasciculation potentials (FPs), a limited number of motor unit potentials (MUPs) from voluntary activity of 12 healthy and seven ALS subjects were also examined. Abnormal propagation of MUPs and scattered IZs were observed in fasciculating units, compared with voluntarily activated MUPs in healthy and ALS subjects. These findings can be related to muscle fiber reinnervation following motor neuron degeneration in ALS and the different origin sites of FPs compared with voluntary MUPs.

## Introduction

**FIGURE 1:**
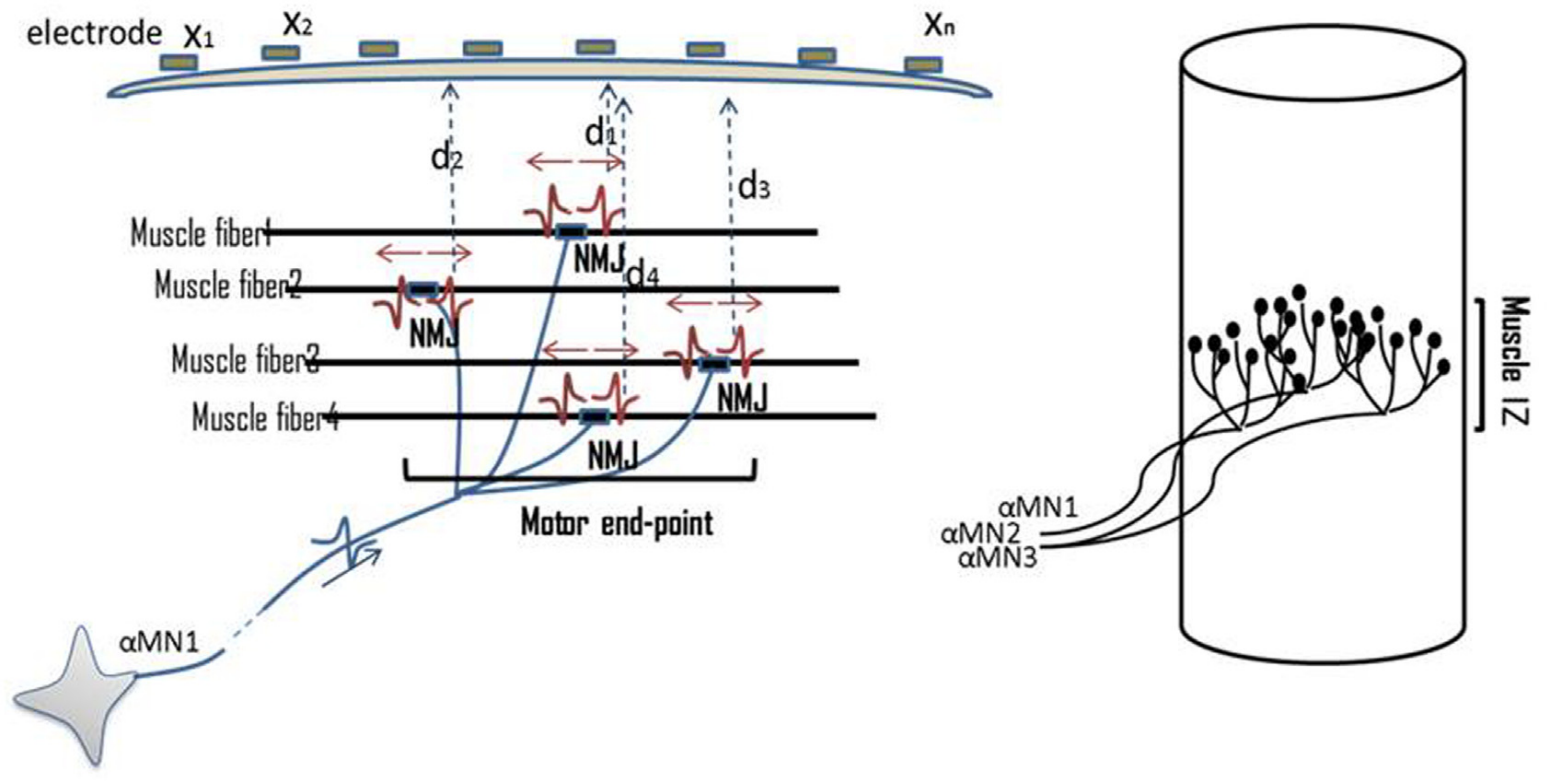
**Illustration of NMJs, motor unit IZ, and muscle IZ band.** Distribution of NMJs along muscle fibers is shown. On top of the skin surface, an array of tiny electrodes is located (*x*_1_–*x*_n_). MUPs are recorded simultaneously on multiple channels. The Multi-channel recording helps to locate the motor unit IZ. The collective IZs from all the motor units will form a muscle’s IZ band.

The IZ in a muscle refers to the site of the muscle and nerve’s connection, i.e., where motor neurons (MNs) and their axons innervate the muscle fibers. Muscle fibers synapse with nerve terminal branches from their MNs at their neuromuscular junctions (NMJs). NMJs are usually located near the mid-point of a muscle fiber, so that excited action potentials on either side travel an approximately equal distance toward the proximal or distal tendons. In this document, the collective site containing the NMJs of a motor unit is called motor unit IZ, and the collective site of all motor unit IZs in a muscle is called muscle IZ band (see **Figure 1**).

The IZ of a muscle can be detected using an array of EMG electrodes placed on the skin surface, such as over the biceps brachii muscle. The biceps IZ is found to have a narrow band at the approximately middle of its longitudinal axis. This has been reported in the 1950s in a healthy population (Coers, 1953; Buchthal et al., 1955). In some pathologic conditions, such as a progressive muscular dystrophy, an enlarged IZ band was reported (Hilfiker and Meyer, 1984). Detection of IZ location has been a subject of interest as an important factor in surface electrode positioning (Brown et al., 1988; Falla et al., 2002; Enck et al., 2004; Martin and MacIsaac, 2006; Mesin et al., 2009a,b), as well as in neurophysiology research to understand muscle morphology and its alterations in diseased states (Lateva et al., 2002).

In this study, we used a linear surface electrode array to record the spontaneous electrical activity of the biceps undergoing active denervation, as seen in patients with ALS, a progressive, degenerative MN disease. Denervation refers to loss of neural drive to a muscle, for example as a result of MN death in ALS. Early after onset of the process, denervation may be followed by the reinnervation process, where disconnected muscle fibers make new synapses with surviving MNs. Evidence of muscle fiber reinnervation is one of the key electrodiagnostic features of ALS.

Fasciculation potentials refer to spontaneous waveforms from a group of muscle fibers comprising a motor unit or multiple units. They may occur in many conditions, including a fatigued muscle or even a normal state muscle. Their occurrence may be prominent in ALS subjects before significant muscle atrophy or weakness has occurred. Detection of fasciculations (from a clinical point of view: muscle twitches) in an anatomic region, its progress in that region, followed by progression into other regions, is considered as a deleterious development for ALS (de Carvalho et al., 2008). While a MUP -initiated by the central drive in voluntary recruitment- originates from a MN in the anterior gray matter of the spinal cord, the site of origin of an FP can be anywhere from the MN’s soma, its axon, or its terminal branches.

The present work differs from previous studies which focused on the frequency of occurrence of FPs (Drost et al., 2007; Kleine et al., 2008, 2012). In this study, we examined the IZ alterations of fasciculating motor units. Technically, determining the location of motor unit IZ by means of multi-channel EMG recording has been used for more than 30 years (Masuda et al., 1983; Falla et al., 2002; Merletti et al., 2003; Mesin et al., 2009a,b).

This study seeks to understand two questions in ALS subjects: (1) whether evidence of scattered NMJs (versus close proximate NMJs seen in normal subjects) is reflected on the corresponding action potential waveform shape and propagation pattern, and (2) whether we can find abnormally enlarged muscle IZ bands in ALS. We addressed these questions by examining the fasciculating motor units from ALS subjects. We also examined MUPs manually extracted from the EMG signal of voluntary activation in both ALS and healthy subjects. As IZ detection is closely related to the pattern of propagation of MUPs along the muscle fibers, we also demonstrated waveforms with abnormal propagations in ALS subjects. In addition, we reported on a preliminary longitudinal study of three of our ALS subjects who had multiple visits.

## Materials and Methods

### Experiments

Electromyographic data were recorded from 12 neurologically intact subjects: six male and six female, aged 20–70 years (average age 43 ± 18 years). We also recruited seven ALS subjects: five male and two female, aged 48–71 years, (average age 56 ± 10 years). The ALS subjects were diagnosed as having “Definite ALS” or “Probable ALS with Laboratory Support” based on El Escorial criteria, (Brooks, 1994). Three ALS subjects had multiple visits, with a total of 15 datasets available from the ALS subjects. **Table 1** provides demographic information about the ALS subjects. This study was approved by the local Human Studies Committee, and all participants gave informed consent before any experimental procedure.

**Table 1:** Demographic information of the ALS subjects.

Subject	Gender	Age (year) at 1st visit	Diagnosis	Age of ALS (in month)			
				Visit1	Visit2	Visit3	Visit4
1	M	56	Definite	11	34	37	41
2	M	71	Probable with Lab Support	23	26	30	33
3	M	46	Definite	37	40	44
4	F	52	Definite	17			
5	M	48	Definite	3			
6	M	52	Definite	28			
7	F	68	Definite	60			

**FIGURE 2:**
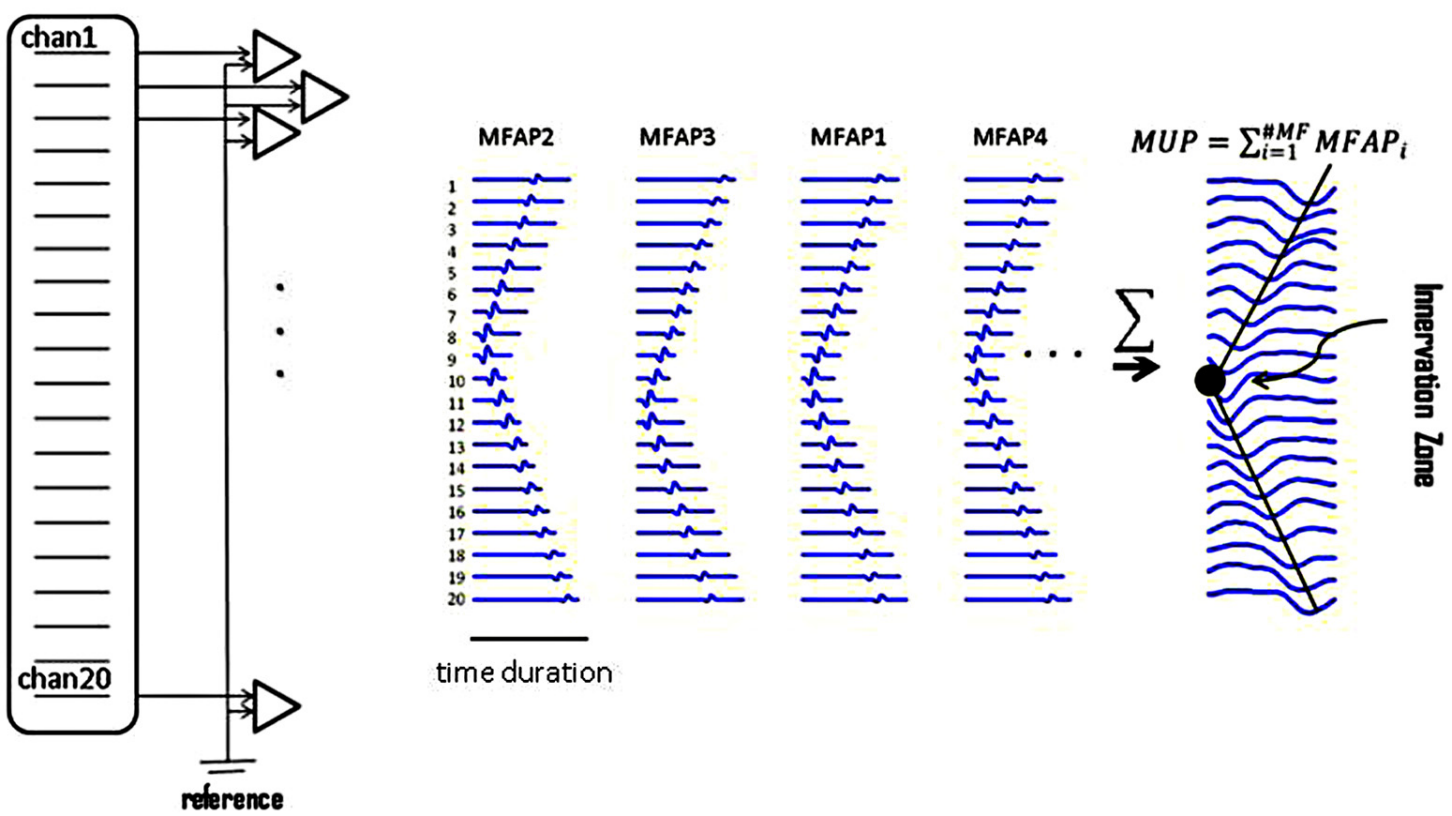
**Raster plots of simulated muscle fiber action potentials (MFAPs) and a final MUP**. This plot shows the concept of MUP generation obtained from summation of single MFAPs on multi-channel electrodes. MFAPs1–4 correspond to muscle fibers 1–4 in **Figure 1**. The IZ can be detected from the multi-channel MUP. In reality, it is difficult to detect single MFAPs from skin surface.

The biceps muscle was examined with the elbow partially flexed and forearm in semi-pronation. A custom-made 20-channel linear bar electrode array (schematic picture in **Figure 2**) was used for all experimental recordings. The IED was 5 mm. Each bar was 1 mm in width and 10 mm in length, arranged in a linear configuration. The electrode array was placed with its center on the belly of the muscle with the long side of the linear bar electrode overlying the long axis of muscle fibers. Channel 1 was on the proximal side and channel 20 covered the distal side. The signals were amplified by the Refa128 EMG Recording System (TMS International BV, The Netherlands). The reference electrode was placed on the ipsilateral elbow. Sampling rate was 2 kHz per channel, with a band pass filter setting at 20–500 Hz.

### Extracting Action Potentials

Multi-channel MUPs were extracted manually from low force levels of voluntary contraction EMG. Irregularly firing FPs (which are less prone to phase interaction or superposition as is commonly seen in MUPs during voluntary activation), were detected automatically using software described by Jahanmiri-Nezhad et al. (2014b). The algorithm first transformed the 20-dimensional signal to a 1-dimensional trace, and applied amplitude thresholding to detect a potential by recording its timing occurrence. The temporal information was then used to extract the multi-channel waveforms from the 20-dimensional signal. Extracted FPs were stored in a database. These waveforms were afterward clustered into classes of similar waveform shapes, by means of the toolbox described by Jahanmiri-Nezhad et al. (2014c).

A waveform in bipolar electrode configuration is most appropriate for IZ analysis. Jahanmiri-Nezhad et al. (2014a) have recently shown that spatial filtering might reduce the sensitivity of FP detection. As a result in this study, many waveforms (especially FPs) were lost in background noise as would be expected; thus, it was impractical to locate their IZ for these motor units.

From voluntary EMG trials, 4–9 different MUPs were manually extracted, from each of the 12 healthy subjects (resulting in a total of 69 different MUPs). The waveforms from each subject were visually examined to ensure they represented different motor units. Ten MUPs were noisy and showed no clear IZ, and thus they were excluded from further analysis. Likewise, 3–9 voluntarily activated motor units (a total of 47 MUPs) were manually extracted from each of nine voluntary EMG recordings of ALS subjects (not all of the 15 recordings from the ALS subjects had voluntary trials). In the seven ALS subjects, 12630 FPs were automatically extracted from 15 trials of long duration recordings (10–30 min). FPs detected from each set of recordings were clustered into a total of 371 classes having similar shapes. Individual waveforms were examined with regard to their motor unit IZ location.

### MUP Simulation; Study of the Effect of NMJ Sites

For illustrative purposes, single MUP waveforms were simulated assuming a 20-channel linear array electrode recording (mimicking our experimental electrode). Similar works can be found in Fuglevand et al. (1992), Roeleveld et al. (1997a,b), Stalberg and Karlsson (2001a,b), McGill (2004), Navallas and Stalberg (2009). The length of the simulated muscle was assumed to be 100 mm. Based on the previous works and the goal of this study, we specified these variables: number of muscle fiber in a motor unit (‘*n*’), the depth of each muscle fiber from the skin surface, its transverse distance from the center of the muscle, the site of its NMJs along the length of the muscle fiber, and longitudinal and radial muscle fiber conductivity. The motor axon and muscle fiber conduction velocities (*v*_n_ and *v*_m_) were assumed 50 m/s and 4–6 m/s (random variable), respectively. The spatial distribution of NMJs in a 3D Cartesian space was modeled by a random variable using a random number generator (mean and SD were determined). Point zero [*x*_0_ = 0, *y*_0_ = 0, *z*_0_ = 0] referred to the single distal branching points on the motor axon. It takes ‘t_nerve_i__’ time for a single action potential to travel from point zero to point [*x*_i_, *y*_i_, *z*_i_], NMJ_i_. Synaptic transmission time was not considered, assuming it had the same value for all NMJs (*i* = 1:*n*). A single action potential was assumed to last for 1 ms.

Variable ‘mfap_0_’ (muscle fiber action potential) denotes a postsynaptic action potential: a 20-sample dipole waveform modeled in Matlab with peak-to-peak amplitude of 17 arbitrary units (au) and a duration of 1 ms, equivalent to a 20 kHz sampling rate. This was later down-sampled to 2 kHz. The mfap_i_resembled the potential corresponding to the NMJ_i_. The amplitude of mfap_i_ decreased as it propagated throughout the volume conductor. The extinction rate was faster when the potential traveled transversely across the muscle, rather than longitudinally along the muscle fiber. Right above the surface of the skin, amplitude was attenuated by a power function of the depth of the muscle fiber from the skin surface. As it propagated along the muscle fiber, it was also attenuated following a power function of the distance traveled. This amplitude attenuation was accompanied with time delays according to muscle fiber conduction velocity, *v*_m_.

Muscle fiber potentials were all summed up to obtain a MUP. Random white noise (amplitude: ±5 au) was added to the signal. At the end, a ‘butterworth’ low pass filter was added as a model of the effect of connective tissue and skin on the signal, with a threshold low-pass frequency of 500 Hz.

### Locating a Motor Unit IZ Based on its Action Potentials

Assume that an array of electrodes covers the longitudinal axis of the biceps. When a motor unit discharges, the channel located immediately over the top of the motor unit IZ site is the one in which the waveform will appear first. The adjacent channels will detect the waveform after a time lag. Propagation of the MUP continues until the amplitude of the MUP waveform attenuates within background noise (an indication that it has reached the end of the muscle fibers). This propagation of the MUP (accompanied with sequential time lags) forms a ‘<’ (letter “v” on its side) shape on the display of the multi-channel waveform, as shown in **Figure 2**. The left-oriented apex of the ‘<’ is considered to be the motor unit’s IZ. Usually, the waveform phase turns opposite around the IZ in bipolar mode.

To facilitate locating the motor unit IZ, a plot of *Points of Extrema* was provided: the points of *maxima* and *minima* – from each channel- were depicted in a single plot. Data in the plot of *Points of Extrema* can be divided into two ‘ascending’ and ‘descending’ sections. The point where these sections meet is the location of the IZ. On the waveform raster plot in bipolar mode, we usually see minimum waveform amplitude. We also see that the phase of waveforms across all channels has a turn at this point. For some waveforms, there may be no time-delay propagation around this point for several IEDs. We propose that such a mechanism may estimate the length of NMJ scatter within that motor unit (through counting the number of channels where no propagation was observed).

### Data Analysis

Individual waveform potentials were visualized in their initial mode with bipolar layout as well as the plotting of *Points of Extrema*. The lengths of the motor unit IZs were estimated in the channels overlying the IZ. If no clear IZ point could be determined, yet there were ascending and descending lines in the plot of *Extrema*, then we estimated the IZ point by extrapolating the lines and locating the point they crossed each other. Muscle IZ band was calculated based on motor unit IZs within a dataset for an individual muscle. Muscle IZ dispersion was calculated as the SD of motor unit IZs within the muscle. To statistically compare the IZ among FPs and MUPs in ALS, and to compare MUPs in ALS and in healthy subjects, we used an un-paired student *t*-test. A Normality test (the Jarque–Bera test built in Matlab) was performed preceding any *t*-test comparison. Where the test of normality failed, Box–Cox transformation ($x^{\prime }=\frac{x^{\lambda }-1}{\lambda }$, *x is the array of positive data and λ is a scalar*) was tried. Box–Cox attempts to remove skewness in a distribution, and make it approximately like a Normal distribution, by searching for a λ leading to the smallest SD in *x*. Nevertheless, a normally distributed *x*′ is not guaranteed, hence the output should always be re-tested.

## Results

### Motor Unit IZ Length and Evidence for Enlarged NMJs’ Scatter

**FIGURE 3:**
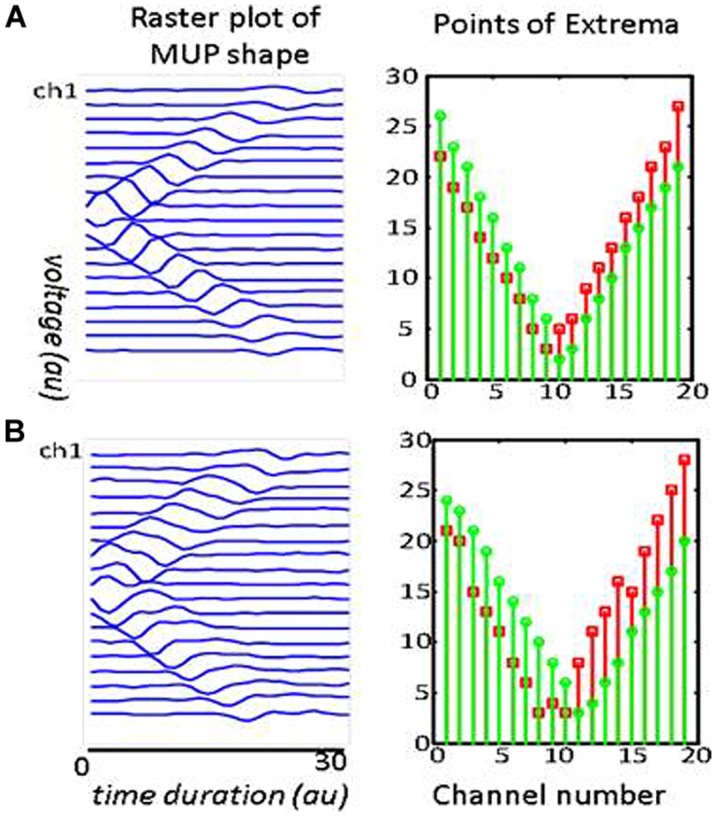
**Two simulated MUPs**. The raster plots are on the left, and corresponding points of extrema are on the right. The sites of minimum are marked with green circles and the maximum with red squares. In the raster plots, the IZ is located at the center (channel 10). The troughs on the right panels occur at *x* = 10. Each MUP is composed of 500 MFAPs. NMJ distribution in **(A)** was spread over a 5 mm range around the center of muscle. The distance between the trough of the points of minima and maxima is consistently about one inter-channel distance (5 mm). NMJ distribution in **(B)** was spread over a 15 mm range. The distance between the troughs’ length is consistently about three inter-channel distance (3 × 5mm = 15 mm).

Visual examination of the raster plot of a waveform, along with the *Points of Extrema*, helped to locate a motor unit IZ. **Figure 3** includes raster plots of two simulated waveforms. The corresponding motor units are identical: they both have 500 innervated fibers and are most superficial to the skin. In **Figure 3B** the NMJs are distributed in a 15 mm segment along the longitudinal axis of the muscle; whereas in **Figure 3A**, they are in a 5 mm segment. The waveform peaks in **Figure 3A** turn a sharp corner, and in **Figure 3B** stay in the same position for approximately the distance between three IEDs, then propagate toward the muscle fiber endings. This simulation aims to illustrate how NMJs scatter affects the corresponding MUP waveform and conceptualize the possibility of measuring the IZ length from the waveform raster plot. It is noted that the resolution in the measurement in this study is one IED equals to 5 mm.

**FIGURE 4:**
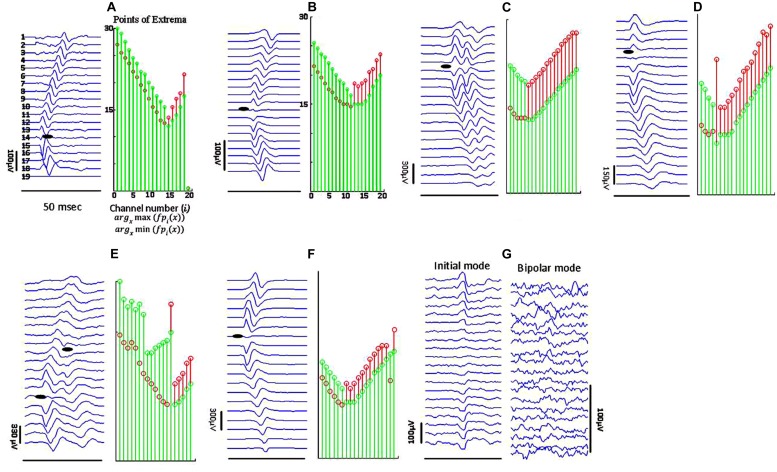
**Sample raster plots (in bipolar mode, unless specified) of experimental data from different subjects, illustrating different propagation patterns with various motor unit IZ length. (A)** Sharp narrow waveforms is likely to represent a superficial motor unit, with narrowly positioned NMJs. **(B)** Smoother waveforms, likely referring to either a motor unit deeper inside the muscle, or corresponding to a thicker subcutaneous layer (between the muscle and skin surface where the electrodes are located). **(C)** Double discharge (commonly observed in ALS subjects). The lines of points of *minima* to *maxima* in descending and ascending line meet at a single point, but they pause for at least four IEDs for the segments of *maxima* to *minima*. In **(D)** there is no time lag for waveforms on channels 3–8. The IZ center is on channel 5. **(E)** Waveform seemingly with two IZ points, most probably from superposition of two FPs. In **(F)** the waveform amplitude attenuates toward the distal channel. **(G)** Abnormal propagation pattern without any time delays across the channels; seen mostly in ALS.

**Figure 4** depicts different types of propagation patterns observed from the experimental data. The first two panels (**Figures 4A,B**) are quite common among all our data sources (voluntary MUPs from healthy and ALS subjects, and FPs from ALS subjects). The waveform raster plots can be categorized into “<,” and “|” patterns as a sign of scattered NMJs in a motor unit, most likely due to reinnervation. We visually estimated motor unit IZ length among 200 fasciculation waveforms in 15 datasets. As shown in **Figures 4A,B** the motor unit IZ length is typically two IEDs. As summarized in **Table 2**, FPs have the largest motor unit IZ lengths, comparing to the MUPs from voluntary trials in healthy and ALS subjects. In comparing ALS and healthy subjects, the former also have larger motor unit IZ length.

**Table 2:** Motor unit IZ Length -measured in millimeters (mm)- from three sources of data: FPs, and MUPs extracted from voluntary trials from healthy and ALS subjects.

FP dataset	Min	Max	Mean	SD/$\sqrt{n}$	SD	MUP _control_	Min	Max	Mean	SD/$\sqrt{n}$	SD
	5	25	14.5	2	6		10	20	13.5	1	3.5
	10	45	17.5	3	11		5	25	12.5	2	5.5
	10	50	17	3	11		10	50	30	7.5	4.7
	10	45	14	2	9		5	15	11.5	1.5	4
	5	25	18	1.7	6.5		5	15	10	2	4
	10	30	17.5	2.5	8		15	25	18.5	1.5	5
	10	40	14	1	7		15	25	20	3	5
	15	25	21	2	4		30	40	35	3	5
	15	35	20.5	2.5	6.5						
	20	20	20	0	0	**MUP_**ALS**_**	**Min**	**Max**	**Mean**	**SD/$\sqrt{n}$**	**SD**
						
	25	25	25	0	0		5	20	13.7	2.5	5
	10	40	20	1.5	8.5		5	15	9	1	3
	15	55	28	4	11.5		15	20	18	1.5	3
	10	30	25	2.5	7		5	15	11.2	2.5	5
	10	65	3.6	5.5	16		10	20	13.5	2	5

				**Averages**		**Min**	**Max**	**Mean**
				
				FPs		12	37	19.5
				MUPs – ALS		12	27	18.9
				MUPs – control		9	18	13

*For each dataset, the minimum, maximum, mean, SD of the mean (SD/$\sqrt{n}$), and SD of motor unit IZ length values are reported. Only datasets containing at least three samples are included.*

### Muscle IZ Band Length; Evidence of Increased Motor Unit IZ Scatter

**FIGURE 5:**
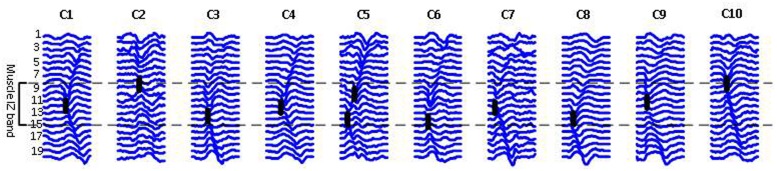
**Raster plots of ten FPs belonging to one ALS subject, each representing a class of waveform shapes**. Class index is reported on the top. C2 has an abnormal straight propagation, with no time lags between the channels. In C5, two IZs were detected, indicating for either two superimposed FPs or a doubly innervated unit as suggested by Lateva et al. (2002, 2010). The heavy black lines indicate IZs.

**Figure 5** shows 10 different FP waveforms from one dataset (one muscle, one trial of EMG recording). Motor unit IZs are marked in bold dark dashes. We define muscle ‘IZ length’ as: the distance between the farthest distal and most proximal points of NMJs located on the muscle fibers, as shown in **Figure 5**. **Table 3** summarizes the result of muscle IZ band measure from our three study populations.

**Table 3:** Measures of muscle IZ band length from FPs compared with MUPs in healthy and ALS subjects.

	MUPs; healthy (12 datasets)	MUPs; ALS (9 datasets)	FPs (15 datasets)
IZ band length (mm): range among datasets	5–25	5–42.5	12.5–75

Average	10.2 ± 8.1	19.2 ± 11.7	39.1 ± 16.3

IZ dispersion (mm): range among datasets	1.4–17.6	2.4–14	4–21

Average	5.1 ± 5	8.7 ± 4.2	12.8 ± 4.9

*Measurements are all in millimeters. Both the IZ band length (distance between the most distal from the most proximal located motor points) and the SD of motor unit IZs within a muscle are reported*.

As shown in **Table 3**, the scatter of muscle IZ length has larger values among MUPs in ALS subjects than in healthy controls. The statistical tests did not confirm its significance though. However, it showed that muscle IZ dispersion (STD) within a muscle in voluntary MUPs of ALS subjects is statistically larger than in healthy subjects (*p* = 6e-5); and that for FPs was larger than for voluntary MUPs from healthy controls and ALS subjects (*p* = 5e-6 and 0.052, respectively).

### Longitudinal Study of Muscle IZ Band

**FIGURE 6:**
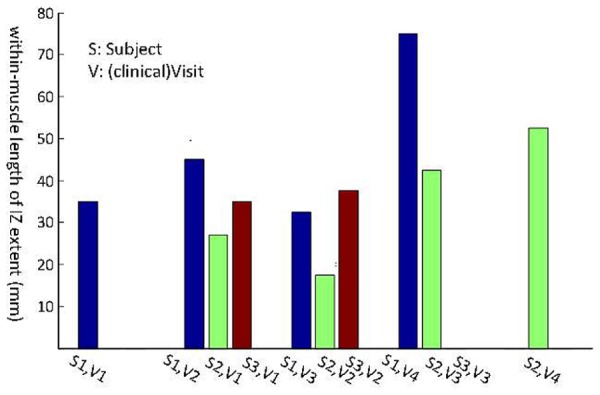
**Longitudinal study of muscle IZ band (from FPs) for three ALS subjects.** Statistical testing was performed on the IZ sites within a muscle. The *x*-axis is the index for each subject (S, Subject and V, Visit).

Three subjects with ALS had multiple visits, as listed in **Table 4**. This allowed us to monitor the changes in their spontaneous discharge activities. Analysis of IZ distribution from these subjects is illustrated in **Figure 6**. In this figure, the bars indicate the extent of IZ band along the muscle. The statistical tests showed significant differences at least on one occasion for all three subjects. Box–cox transformation was needed for Subject 2, with ‘λ = 1.5.’ The not available (NA) length of IZ for the last visit of Subject 3, was the result of a very weakened muscle with only few classes of FPs that had no clear IZ.

**Table 4:** Longitudinal data of muscle IZ band length (from FPs) in millimeters.

IZ length (mm)	July 2009	June 2011	August 2011	December 2011	March 2012
Subject1	35	45	32.5	75	–
Subject2	–	27.5	17.5	42.5	52.5
Subject3	–	35	37.5	NA	–

## Discussion

This study aimed to investigate –in a non-invasive manner– the distribution of NMJs along the longitudinal axis of the fasciculating motor units of biceps in ALS. We specifically defined and measured motor unit IZ and muscle IZ band. Based on our definitions, we observed abnormally enlarged fasciculating motor unit IZ length and muscle IZ scatter than what has been previously published for neurologically intact biceps. Usually a waveform has a ‘<’ shape along muscle fibers on its raster plot. In a bipolar configuration, at the tip point –assumingly the IZ point– we can observe minimum amplitude waveform and a phase turn. We found that the dispersion of IZs within a muscle based on the FPs was larger than that of voluntary MUPs from ALS, and larger in ALS than in healthy control subjects. Scatter of IZ points within a muscle ranged 5–25 mm among control, 5–42.5 mm among ALS (from voluntary MUPs), and 12.5–75 mm among ALS (from FPs), which is larger than previously published values (10–20 mm reported in Masuda et al., 1983; 14 mm reported in Masuda and Sadoyama, 1986). We also examined the longitudinal data from the three ALS subjects, with multiple visits every 3 months on average. A significant increase of IZ dispersion was observed for at least once between the visits, for all the three subjects.

In this study, we hypothesized – based on a simulation study– that scatter of NMJs in a motor unit can be reflected on its waveform raster plot. The IZ turning point (or width) usually happens at around 1–3 IED(s). This value ranged on average 1.8–3.6 IEDs among MUPs of control subjects, 2.4–5.4 IEDs among MUPs of ALS subjects, and 2.4–7.4 IEDs among FPs in ALS. Therefore, we observed larger NMJs scatter in motor units of ALS subjects.

We defined motor unit IZ as the collective site of NMJs within a motor unit, and defined muscle IZ band as the collective site of motor unit IZs in a muscle. IZ and motor point both generally refer to the site of the motor nerve terminal connection to muscle fibers. The IZ has been traditionally defined physiologically using a surface recording electrode array or morphologically by histochemistry. The motor point of a muscle is conventionally determined as the focal point of supramaximal electrical stimulation of a motor nerve resulting in the largest evoked response (Guzman-Venegas et al., 2014).

Innervation zone distribution within biceps has been previously reported to have a narrow band in healthy subjects (Masuda et al., 1983; Masuda and Sadoyama, 1986; Brown et al., 1988; DeFreitas et al., 2010; Barbero et al., 2012). Aquilonius et al. (1984) applied cholinesterase staining and showed the NMJs forming distinct V shaped bands in the middle of biceps (<10 mm). The NMJs were also observed in more distal sites.

Increased variation of the location of IZs in subjects with ALS can be an indicator of ongoing reinnervation process in a muscle under denervation. When a MN dies, its corresponding muscle fibers become ‘orphaned,’ which may consequently be ‘adopted’ by surviving motor units. This results in a collateral sprouting from the terminal motor axon branches (reinnervation). More scattered IZ might be due to the reinnervation process where the added myoneural junctions expand the length of IZ.

The origin site of excitation of an FP might be another relevant factor contributing to the abnormal IZ locations observed in ALS. FPs might originate from anywhere of the MN soma, its axon, or at the terminal branches. Furthermore, FPs may be originated from a group of several motor units (de Carvalho and Swash, 2012). This may cause observation of enlarged motor unit IZs.

In estimating IZ site based on EMG, electrode location is a key factor. In this work, all the FPs were extracted from a single trial of EMG recording from a completely relaxed muscle. Hence, the possibility of a shift in IZ location due to muscle contraction (as described in Martin and MacIsaac, 2006; Piitulainen et al., 2009; Nishihara et al., 2010, 2013) was excluded. One limitation of the study was that only a 1-dimensional linear electrode array was used. Hence, analysis of medial–lateral IZ distribution was not available (which requires 2-dimensional electrode array recording). Furthermore, we analyzed IZ location and length partially based on visual examination versus a fully automated analysis. Currently, a fully automated software cannot achieve comparable performance to a human expert visual analysis due to the wide range of different cases of MUP and fasciculation propagation patterns. In our study, we observed that it is difficult to generalize a certain model of propagation applied to all the experimental data. Fully automated software may provide a more reproducible result, but not necessarily an more accurate one.

Finally, it is worth to mention that we measured motor unit IZ length by counting channels on which the initial waveform does not propagate, a means to measure NMJ scatter. However, parameters such as motor unit depth, subcutaneous layer thickness, and noise may complicate this measurement, as mentioned in Barkhaus and Nandedkar (1994) and Barkhaus et al. (2006). As the distance between the generator source and the sensor increases, the high frequency content of the signal is attenuated. Comparing the waveforms in **Figure 4** is a relevant example where panel (**Figure 4A**) might represent a more superficial motor unit and (**Figure 4B**) a deeper one. One reason justifying this claim is the smoothed waveform shapes in panel (**Figure 4B**). We observed many of long IZ length (around 2–3 IEDs), belong to this category. Nonetheless, waveforms such as in same figure panel (**Figure 4C**), are likely to be from reinnervated units, and were observed more in ALS data as our results depicted. Further systematic simulation work is needed to investigate the effect of different (both physiologic and non-physiologic) factors that may affect waveform potentials, and to better understand the experimental recordings.

## Conflict of Interest Statement

The authors declare that the research was conducted in the absence of any commercial or financial relationships that could be construed as a potential conflict of interest.

## Abbreviations

ALS: amyotrophic lateral sclerosis
EMG: electromyogram
FP: fasciculation potential
IED: inter electrode distance
IZ: innervation zone
MN: motor neuron
MUP: motor unit potential
NMJ: neuromuscular junction
